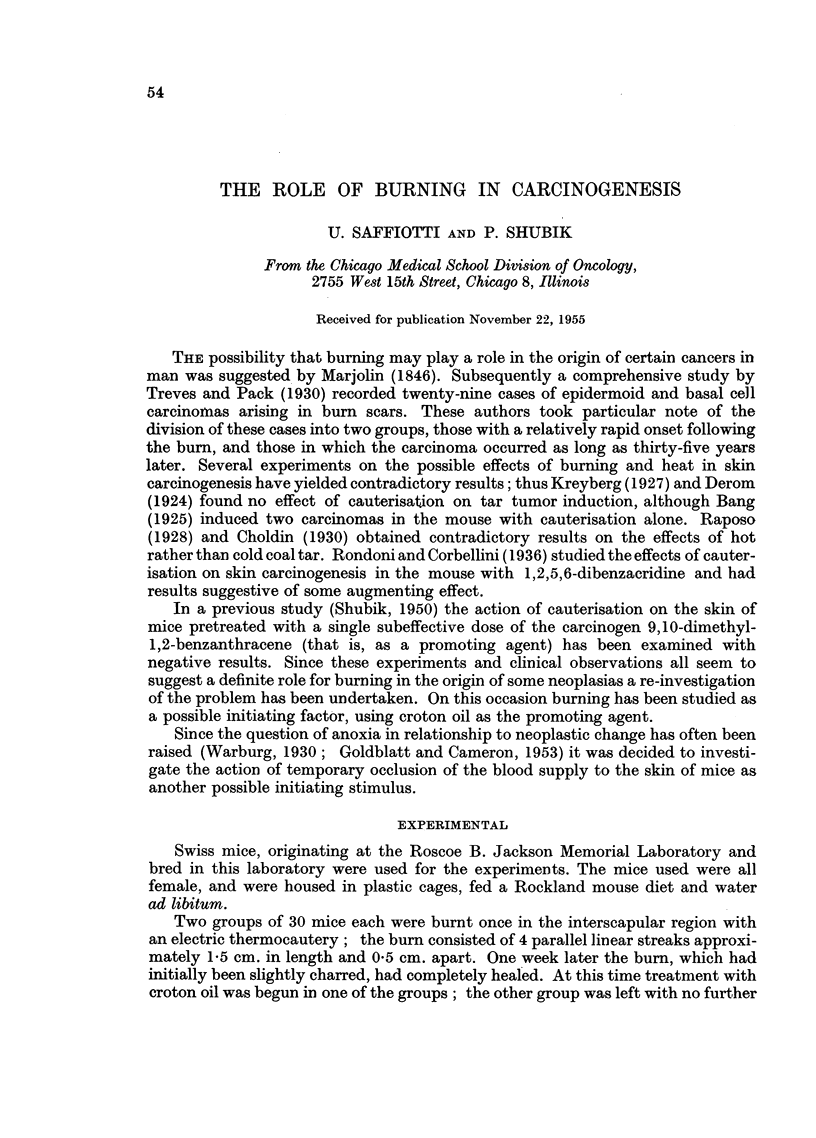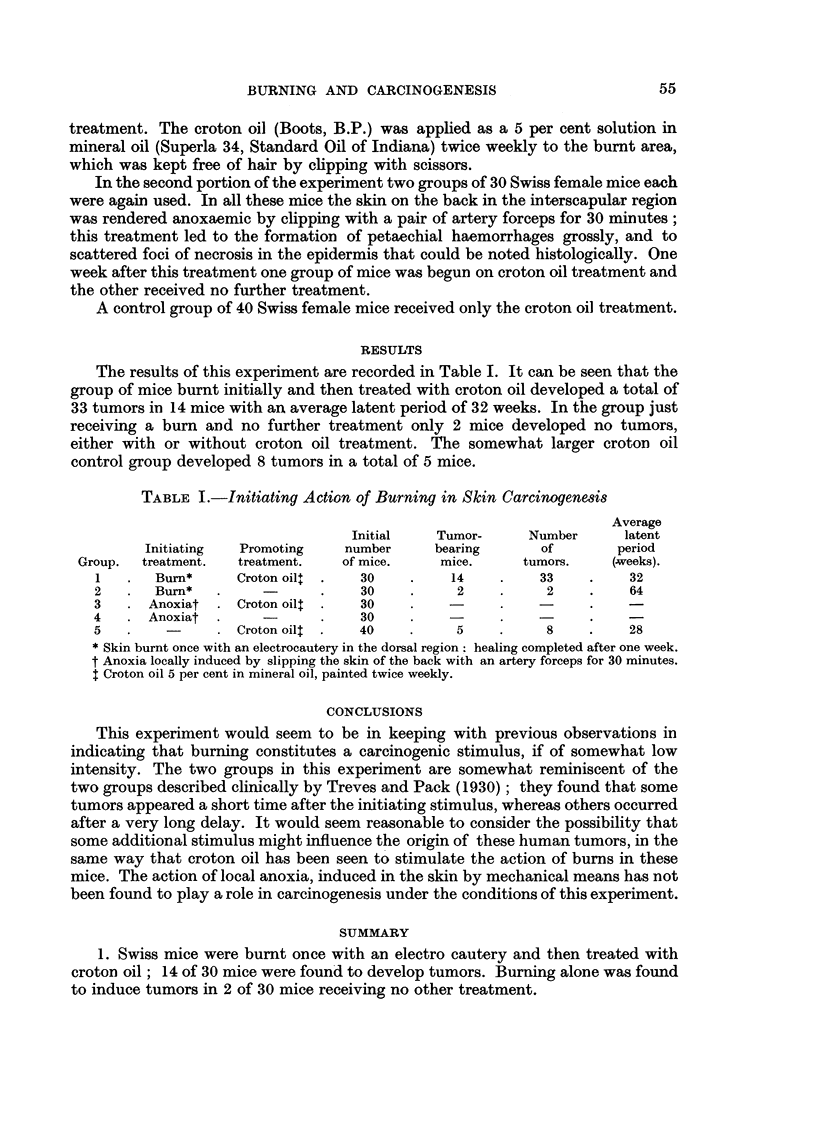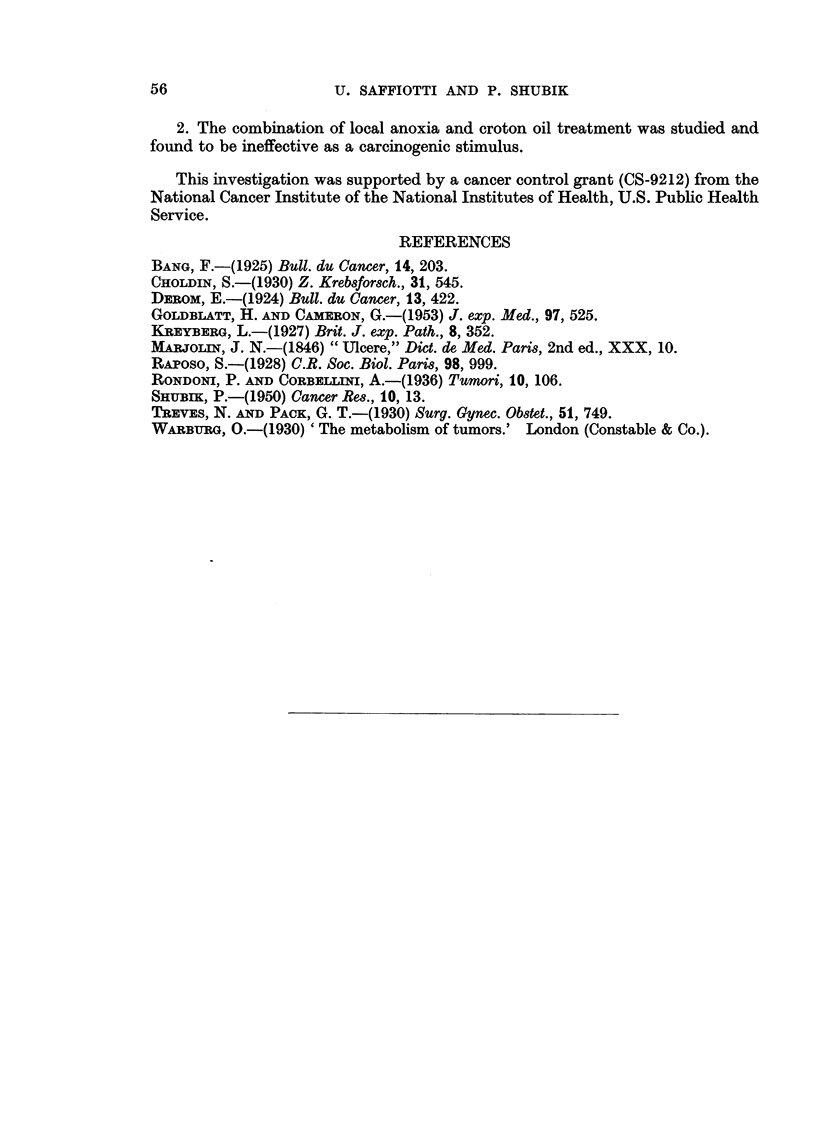# The Role of Burning in Carcinogenesis

**DOI:** 10.1038/bjc.1956.7

**Published:** 1956-03

**Authors:** U. Saffiotti, P. Shubik


### THE ROLE OF BURNING IN CARCINOGENESIS

U. SAFFIOTTI AND P. SHUBIK

*From the Chicago Medical School Division of Oncology,*
*2755 West 15th Street, Chicago 8, Illinois*

Received for publication November 22, 1955

THE possibility that burning may play a role in the origin of certain cancers in man was suggested by Marjolin (1846). Subsequently a comprehensive study by Treves and Pack (1930) recorded twenty-nine cases of epidermoid and basal cell carcinomas arising in burn scars. These authors took particular note of the division of these cases into two groups, those with a relatively rapid onset following the burn, and those in which the carcinoma occurred as long as thirty-five years later. Several experiments on the possible effects of burning and heat in skin carcinogenesis have yielded contradictory results; thus Kreyberg (1927) and Derom (1924) found no effect of cauterisation on tar tumor induction, although Bang (1925) induced two carcinomas in the mouse with cauterisation alone. Raposo (1928) and Choldin (1930) obtained contradictory results on the effects of hot rather than cold coal tar. Rondoni and Corbellini (1936) studied the effects of cauterisation on skin carcinogenesis in the mouse with 1,2,5,6-dibenzacridine and had results suggestive of some augmenting effect.

In a previous study (Shubik, 1950) the action of cauterisation on the skin of mice pretreated with a single subeffective dose of the carcinogen 9,10-dimethyl-1,2-benzanthracene (that is, as a promoting agent) has been examined with negative results. Since these experiments and clinical observations all seem to suggest a definite role for burning in the origin of some neoplasias a re-investigation of the problem has been undertaken. On this occasion burning has been studied as a possible initiating factor, using croton oil as the promoting agent.

Since the question of anoxia in relationship to neoplastic change has often been raised (Warburg, 1930; Goldblatt and Cameron, 1953) it was decided to investigate the action of temporary occlusion of the blood supply to the skin of mice as another possible initiating stimulus.

### EXPERIMENTAL

Swiss mice, originating at the Roscoe B. Jackson Memorial Laboratory and bred in this laboratory were used for the experiments. The mice used were all female, and were housed in plastic cages, fed a Rockland mouse diet and water *ad libitum.*

Two groups of 30 mice each were burnt once in the interscapular region with an electric thermocautery; the burn consisted of 4 parallel linear streaks approximately 1·5 cm. in length and 0·5 cm. apart. One week later the burn, which had initially been slightly charred, had completely healed. At this time treatment with croton oil was begun in one of the groups; the other group was left with no further treatment. The croton oil (Boots, B.P.) was applied as a 5 per cent solution in mineral oil (Superla 34, Standard Oil of Indiana) twice weekly to the burnt area, which was kept free of hair by clipping with scissors.

In the second portion of the experiment two groups of 30 Swiss female mice each were again used. In all these mice the skin on the back in the interscapular region was rendered anoxaemic by clipping with a pair of artery forceps for 30 minutes ; this treatment led to the formation of petaechial haemorrhages grossly, and to scattered foci of necrosis in the epidermis that could be noted histologically. One week after this treatment one group of mice was begun on croton oil treatment and the other received no further treatment.

A control group of 40 Swiss female mice received only the croton oil treatment.

### RESULTS

The results of this experiment are recorded in Table I. It can be seen that the group of mice burnt initially and then treated with croton oil developed a total of 33 tumors in 14 mice with an average latent period of 32 weeks. In the group just receiving a burn and no further treatment only 2 mice developed no tumors, either with or without croton oil treatment. The somewhat larger croton oil control group developed 8 tumors in a total of 5 mice.

TABLE I.—*Initiating Action of Burning in Skin Carcinogenesis*

| Group. | Initiating treatment. | Promoting treatment. | Initial number of mice. | Tumor-bearing mice. | Number of tumors. | Average latent period (weeks). |
|---|---|---|---|---|---|---|
| 1 | Burn* | Croton oil‡ | 30 | 14 | 33 | 32 |
| 2 | Burn* | — | 30 | 2 | 2 | 64 |
| 3 | Anoxia† | Croton oil‡ | 30 | — | — | — |
| 4 | Anoxia† | — | 30 | — | — | — |
| 5 | — | Croton oil‡ | 40 | 5 | 8 | 28 |

* Skin burnt once with an electrocautery in the dorsal region : healing completed after one week.
† Anoxia locally induced by slipping the skin of the back with an artery forceps for 30 minutes.
‡ Croton oil 5 per cent in mineral oil, painted twice weekly.

### CONCLUSIONS

This experiment would seem to be in keeping with previous observations in indicating that burning constitutes a carcinogenic stimulus, if of somewhat low intensity. The two groups in this experiment are somewhat reminiscent of the two groups described clinically by Treves and Pack (1930) ; they found that some tumors appeared a short time after the initiating stimulus, whereas others occurred after a very long delay. It would seem reasonable to consider the possibility that some additional stimulus might influence the origin of these human tumors, in the same way that croton oil has been seen to stimulate the action of burns in these mice. The action of local anoxia, induced in the skin by mechanical means has not been found to play a role in carcinogenesis under the conditions of this experiment.

### SUMMARY

1. Swiss mice were burnt once with an electro cautery and then treated with croton oil ; 14 of 30 mice were found to develop tumors. Burning alone was found to induce tumors in 2 of 30 mice receiving no other treatment.

2. The combination of local anoxia and croton oil treatment was studied and found to be ineffective as a carcinogenic stimulus.

This investigation was supported by a cancer control grant (CS-9212) from the National Cancer Institute of the National Institutes of Health, U.S. Public Health Service.

### REFERENCES

Bang, F.—(1925) *Bull. du Cancer*, **14**, 203.
Choldin, S.—(1930) *Z. Krebsforsch.*, **31**, 545.
Derom, E.—(1924) *Bull. du Cancer*, **13**, 422.
Goldblatt, H. and Cameron, G.—(1953) *J. exp. Med.*, **97**, 525.
Kreyberg, L.—(1927) *Brit. J. exp. Path.*, **8**, 352.
Marjolin, J. N.—(1846) " Ulcere," *Dict. de Med. Paris*, 2nd ed., XXX, 10.
Raposo, S.—(1928) *C.R. Soc. Biol. Paris*, **98**, 999.
Rondoni, P. and Corbellini, A.—(1936) *Tumori*, **10**, 106.
Shubik, P.—(1950) *Cancer Res.*, **10**, 13.
Treves, N. and Pack, G. T.—(1930) *Surg. Gynec. Obstet.*, **51**, 749.
Warburg, O.—(1930) ' The metabolism of tumors.' London (Constable & Co.).